# OLDER VETERANS’ PERSPECTIVES ON MEASURING FUNCTIONAL STATUS IN VA PRIMARY CARE CLINICS

**DOI:** 10.1093/geroni/igz038.2773

**Published:** 2019-11-08

**Authors:** Francesca Nicosia, Malena J Spar, Alicia Neumann, Molly C Silvestrini, Maureen Barrientos, Rebecca T Brown

**Affiliations:** 1 San Francisco VA Health Care System, San Francisco, California, United States; 2 San Francisco VA Medical Center Division of Geriatrics; San Francisco, California, United States; 3 Division of Geriatrics, San Francisco, California, United States; 4 Corporal Crescenz VA Medical Center Division of Geriatric Medicine, Philadelphia, Pennsylvania, United States

## Abstract

Little is known about older adults’ perspectives on measuring functional status (i.e., ability to perform basic and instrumental activities of daily living). This study used a qualitative design to understand older Veterans’ perspectives on measuring function in primary care settings. Thematic analysis of interviews conducted with 28 Veterans ≥65 years and 5 caregivers from one VA Medical Center identified several themes including: 1) importance and relevance of discussing function; 2) preferences for assessment method (e.g., provider- or self-assessment;) and 3) wording of questions (i.e., needing help vs. having difficultly). These findings suggest that effective approaches to measuring function must consider patient preferences on content and format and ensure that measurement is used to inform care. We applied these findings to develop an interprofessional intervention to improve functional status measurement for older Veterans in primary care.

